# A 2-Year Field Study Shows Little Evidence That the Long-Term Planting of Transgenic Insect-Resistant Cotton Affects the Community Structure of Soil Nematodes

**DOI:** 10.1371/journal.pone.0061670

**Published:** 2013-04-16

**Authors:** Xiaogang Li, Biao Liu

**Affiliations:** 1 Nanjing Institute of Environmental Sciences, Ministry of Environmental Protection of China, Nanjing, Jiangsu Province, China; 2 Key Laboratory of Soil Environment and Pollution Remediation, Institute of Soil Science, Chinese Academy of Sciences, Nanjing, Jiangsu Province, China; French National Institute for Agricultural Research (INRA), France

## Abstract

Transgenic insect-resistant cotton has been released into the environment for more than a decade in China to effectively control the cotton bollworm (*Helicoverpa armigera*) and other Lepidoptera. Because of concerns about undesirable ecological side-effects of transgenic crops, it is important to monitor the potential environmental impact of transgenic insect-resistant cotton after commercial release. Our 2-year study included 1 cotton field where non-transgenic cotton had been planted continuously and 2 other cotton fields where transgenic insect-resistant cotton had been planted for different lengths of time since 1997 and since 2002. In 2 consecutive years (2009 and 2010), we took soil samples from 3 cotton fields at 4 different growth stages (seedling, budding, boll-forming and boll-opening stages), collected soil nematodes from soil with the sugar flotation and centrifugation method and identified the soil nematodes to the genus level. The generic composition, individual densities and diversity indices of the soil nematodes did not differ significantly between the 2 transgenic cotton fields and the non-transgenic cotton field, but significant seasonal variation was found in the individual densities of the principal trophic groups and in the diversity indices of the nematodes in all 3 cotton fields. The study used a comparative perspective to monitor the impact of transgenic insect-resistant cotton grown in typical ‘real world’ conditions. The results of the study suggested that more than 10 years of cultivation of transgenic insect-resistant cotton had no significant effects–adverse or otherwise–on soil nematodes. This study provides a theoretical basis for ongoing environmental impact monitoring of transgenic plants.

## Introduction

Many crops have been transformed to provide enhanced resistance against pests and diseases. Crops expressing δ-endotoxins of *Bacillus thuringiensis* (*Bt*) active against Lepidopteran and Coleopteran insect pests are the most widely grown [1]. The global area of transgenic crops has increased from 1.7 million hectares in 1996 to 160 million hectares in 2011 [1]. Transgenic insect-resistant cotton expressing Cry1Ab/c and/or CpTI (Cowpea Trypsin Inhibitor) has been released into the environment for commercial cultivation for more than a decade in China. Its planted area currently represents 71.5% of the total cotton grown in China [1]. These lines effectively control cotton bollworm and other Lepidoptera, resulting in a significant reduction in the usage of chemical insecticides, thus protecting the environment and human health while yielding substantial socioeconomic benefits [2]–[4]. Nevertheless, as with any technology, there have been questions about the potential environmental risks associated with transgenic plants. One of the major ecological concerns about the environmental risks of transgenic insect-resistant plants is the potential effects of these plants on non-target organisms [5]–[7].

The effects of transgenic insect-resistant cotton on non-target pests and natural enemies have been extensively assessed [8], [9]. Most studies have found no convincing and meaningful negative effects of transgenic insect-resistant cotton on the population density, abundance, species richness and diversity of non-target arthropod natural enemies [9]–[13]. Pollinators are also important non-target organisms, and the impacts of transgenic crops on these organisms have also been evaluated. Feeding tests, as well as field surveys, have been extensively performed to evaluate the safety of *Bt* plants for honey bees or pollinating beetles, and no significant adverse effects on longevity, feeding and learning behavior, the development of the hypopharyngeal glands or superoxide dimutase activity have been observed in these insects [14]–[21].

Transgenic insect-resistant cotton could also affect soil organisms. Transgenic proteins, such as Cry1Ab and Cry1Ac, can be released into the soil from cotton residues, root exudates and pollen during growth and after harvest [22], [23]. Once in the soil, the toxins can be bound to clay and humus particles [24]. This state protects them from biodegradation and preserves their insecticidal activity [25], and it may pose a potential, inadvertent risk to soil-dwelling organisms [5], [26]. Free-living soil nematodes are the most abundant and species-rich metazoan group in soils [27]. Nematodes are useful indicators of soil quality because of their great diversity and participation in many functions at different levels of the food webs in the soil. They are relatively simple to separate and enumerate. Their populations, in contrast to those of bacteria, are stable in response to changes in soil moisture and temperature [28]. Impacts on soil nematodes are, therefore, an important aspect of the environmental risk assessment and post-release monitoring of transgenic insect-resistant plants.

Certain *Bt* toxins, e.g., Cry5B, Cry6A, Cry14A and Cry21A, have been found to have direct toxic effects on some nematode species [29]. Nevertheless, Cry1Ac and CpTI expressed simultaneously by transgenic crops have not been evaluated for their effects on nematodes. To date, studies on the effects of transgenic insect-resistant plants, such as those expressing Cry1Ab, on soil nematodes have produced contrasting results. Negative effects of Cry1Ab protein on the growth, number of eggs and reproduction of soil nematodes have been detected in the rhizosphere soil of transgenic Cry1Ab maize [30]. However, the results of laboratory and field studies have generally shown no consistent effects of transgenic insect-resistant plants on soil nematodes [31]–[34]. For example, no significant differences in the numbers, communities and biodiversity of nematodes have been found in the soil of maize expressing the Cry1Ab protein relative to non-*Bt* maize [31], [32]. A significant but transient decrease in the numbers of nematodes in soil under *Bt* maize expressing the Cry1Ab protein at 3 different field sites was found in a comparison with non-*Bt* maize, whereas studies conducted in a greenhouse showed no toxic effects of the Cry1Ab protein on populations of nematodes [35], [36]. The reasons for the differences between the 2 studies are unclear, but they may have resulted from different environmental conditions in the greenhouse and the field, which could affect the interactions between plants and soil organisms.

As the results of these studies only reflect short histories of transgenic cultivation, we still face considerable gaps in our scientific understanding of longer-term community-level impacts on soil nematodes from the cultivation of transgenic insect-resistant crops [37]. The biosafety regulations for genetically modified organisms (GMOs) in many jurisdictions, including both China and the European Union (EU), require monitoring of environmental impacts after the environmental release and commercial cultivation of transgenic crops [38], [39]. Transgenic insect-resistant cottons have now been planted for more than a decade in China, a nation prominent in pioneering the use of this new technology. The cotton fields of China therefore offer a valuable opportunity to address scientific questions of longer-term impacts and to fulfill the ongoing demands of biosafety regulations. The purpose of this study was to investigate the population density and community structure of soil nematodes over 2 years, comparing the soil nematodes of a conventional non-transgenic cotton plantation with those from soils that have been planted with transgenic cotton expressing Cry1Ab/c and CpTI for up to 10 years, and to provide a theoretical basis for environmental impact monitoring of transgenic plants.

## Materials and Methods

### Plant Material and Field Trial

This study was conducted in fields at a cotton farm in Baibi town, Anyang, Henan Province, China. The farm belongs to the Cotton Research Institute (CRI) of the Chinese Academy of Agricultural Sciences (CAAS). This field site is in the North Temperate Zone and has a continental monsoon climate. The annual mean temperature is 13.6 °C, and the annual mean precipitation is 606.1 mm. Three types of cotton fields were selected for this study (Table 1). Field T-1 was originally planted with non-transgenic cotton, then sown beginning in 2002 with the transgenic insect-resistant cotton line Zhong-41 expressing Cry1Ab/c and CpTI. Field T-2 was planted with 2 transgenic Cry1Ab/c cotton lines, Zhong-29 and Zhong-30, from 1999 to 2001 and subsequently planted with Zhong-41 beginning in 2002. Field CK has been planted with the conventional non-transgenic cotton line Zhong-35 since 1999. Zhong-29 and Zhong-30 were developed by CRI, CAAS, and approved by the Ministry of Agriculture (MOA) of China in 1998. Zhong-41 was developed jointly by CRI and the Biotechnology Research Institute of CAAS and approved by the MOA in 2002 [40]. The content of Cry1Ab/c expressed in the leaves of Zhong-41, Zhong-29 and Zhong-30 was determined, and the results indicated that the transgene expression remained stable during the survey period (Table 1).

**Table 1:** Planting information for 3 cotton fields.

Treatments (Cotton fields)	Cotton lines	Transgenes	Contents of Cry1Ab/c (ng •g^−1^ fresh tissue)	Planting time
CK	Zhong-35	_	_	1999 to 2010
T-1	Zhong-41	Cry1Ab/c# & CpTI	150–600	2002 to 2010
T-2	Zhong-29 & Zhong-30	Cry1Ab/c	200–550	1999 to 2001
	Zhong-41	Cry1Ab/c & CpTI	150–600	2002 to 2010

# Cry1Ab/c represents a fusion gene of Cry1Ac and Cry1Ab.

**Figure 1:**
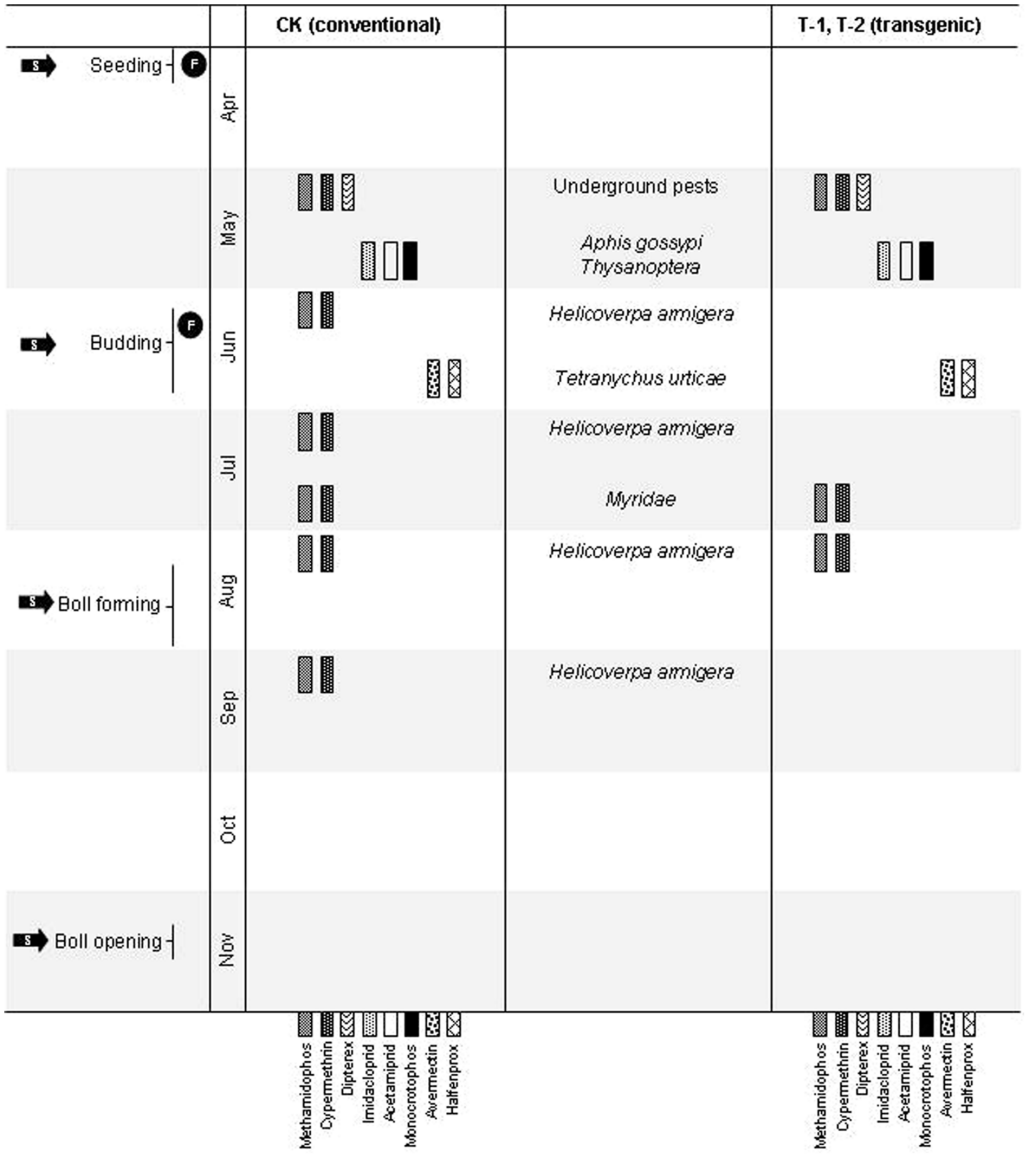
Schematic of agricultural practices applied to the 3 cotton fields during this study (2007–2010). Patterned bars represent different pesticides, with target pest listed in central column. Black circles with ‘F’ indicate fertilizer application, and black arrows with ‘S’ represent sampling times. Symbol placement is indicative of timing but is not precise.

The 3 cotton fields monitored were all located at 36° 7′ N and 116° 22′ E and were composed of a cambisol-type soil (FAO (1998) classification) with the following properties (on a dry mass basis): pH (soil: water ratio 1∶2.5) 7.82, organic C 16.10 g•kg^−1^, total *N* 0.84 g•kg^−1^, total *P* 0.85 g•kg^−1^, total *K* 7.61 g•kg^−1^, available *P* 26.62 mg•kg^−1^, available *K* 134.47 mg•kg^−1^ and soil clay (<0.002 mm) 9.29%. The fields CK, T-1 and T-2 were distributed side by side from south to north and separated by belts 50 m wide. The cotton growing season extended from April to November annually. The agricultural practice for the cotton in the 3 fields was the same as that used for conventional cottons. Fertilizer was applied at the seedling stage and at the budding stage. During the growing seasons, chemical pesticides were used for pest control as necessary (Fig. 1). In addition, all 3 fields lay fallow from November to the following April.

### Soil Sample Collection

Soil samples were collected 4 times per year from 2009 to 2010, inclusive, coinciding with the major growth stages of cotton, namely, seedling (April), budding (June), boll-forming (August) and boll-opening (November). Fifteen meters were left at both ends of every treatment to eliminate marginal field effects on soil sampling. Each type of treatment field was established in triplicate, and the plot size for each replication was 0.17 hectare. One soil sample was collected between 0 and 20 cm deep with 5 cores using a soil auger with a 4 cm diameter and then placed in a sterile plastic bag. Three soil samples were taken in each replication according to the checkerboard method [41]. The soil samples were immediately transported to the laboratory to isolate the soil nematode specimens. The data from 3 soil samples in each replication were pooled, and 3 replicates from each field were used in further statistical analyses.

### Extraction and Identification of Nematodes

Nematodes were extracted from fresh soil equivalent to 100 g dried soil with a sieving process followed by sugar flotation [42]. The nematodes were heat killed and fixed in 4% formaldehyde. They were then counted under a dissecting microscope at 25× magnification. A total of 100 specimens per sample were then randomly selected and identified to the genus level, as described in Liang et al. (2009), at 200× magnification using an inverted compound microscope [43].

### Nematode Community Analysis

Nematode abundances were ln (x +1) transformed prior to statistical analysis and expressed as numbers per 100 g dry soil. Nematode biodiversity was measured with the Simpson and Shannon–Wiener diversity indices. Both indices are sensitive to the abundance of the most common/dominant species in a population [44]. Simpson’s index is defined by the equation 
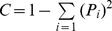
. The Shannon–Wiener diversity index is defined by the equation 
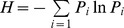
. In these equations, *P_i_* denotes the proportion of soil nematode individuals in each treatment; *P_i_* = N_i_/N, where N_i_ is the abundance of the *i* ^th^ species and N is the overall total abundance in each treatment. *P_i_*≥10% represents dominant groups, 10%>*P_i_*≥1% represents common groups and *P_i_*<1% represents rare groups.

Nematode taxa were ranked along a colonizer–persister (c–p) scale of 1–5 according to Bongers and Ferris (1999) [45]. The Maturity Index (MI) was calculated using the equation of Bongers (1990) [46]:
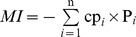
, where P_i_ is the frequency of the taxon in the sample and cp_i_ is the c-p value of taxon i.

Nematode taxa were classified into 5 main trophic groups: bacterial feeders, fungal feeders, plant parasites, omnivores and predators [47]. Based on the c–p and feeding type classification, nematode taxa were also categorized in functional guilds according to Ferris *et al.* (2001): Ba_n_, Fu_n_, Ca_n_, and Om_n_ = bacterial feeders, fungal feeders, predators, and omnivores, respectively, with n = c–p value [48]. The following indices were calculated to describe the enrichment and structure conditions as well as the predominant decomposition channels in the soil food webs: Enrichment inde**x**: 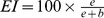, Structure index: 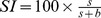, Channel index: *CI* = 100(0.8 Fu_2_)/(3.2 Ba_1_+0.8 Fu_2_), where b = (Ba_2_+ Fu_2_) × 0.8, e = Ba_1_ × 3.2+ Fu_2_ × 0.8, and s = Ca_2_ × 0.8+ (Ba_3_+Ca_3_+Fu_3_+Om_3_) × 1.8+ (Ba_4_+Ca_4_+Fu_4_+Om_4_) × 3.2+ (Ba_5_+Ca_5_+Fu_5_+Om_5_) × 5.

The response of the soil nematode community to the factors ‘treatment’ and ‘sampling time’ was examined with a 2-way ANOVA (Proc GLM). Significance was measured at the alpha = 0.05 level. Principal component analysis (PCA), a repeated-measures multivariate ordination analysis, was performed to identify the influence of treatment and sampling time on community structure (SPSS 13.0 for Windows). The principal components whose eigenvalue exceeded 1 were selected for the analysis.

## Results

### The Composition of the Soil Nematode Community in the 3 Cotton Fields

**Figure 2:**
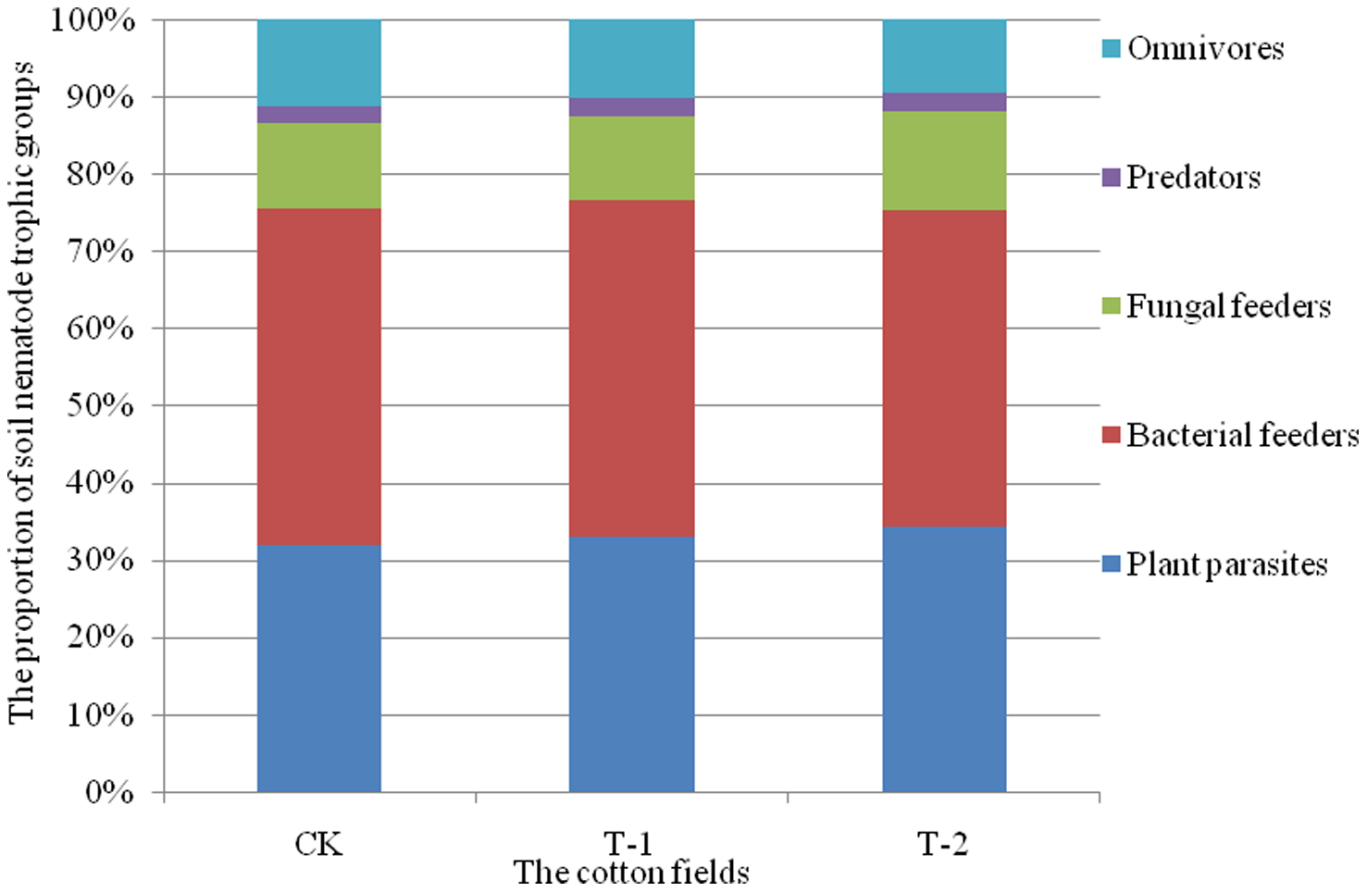
The soil nematode composition in 3 cotton fields during 2009–2010.

Twenty-eight genera of soil nematodes were identified in the 3 cotton fields during different cotton growth stages over 2 years (Table 2). The overall results showed that the most abundant common groups in CK, T-1 and T-2 were *Helicotylenchus*, *Filenchus* and *Acrobeloides*. Most of the genera collected from the soil, such as *Tylenchus*, *Pratylenchus*, *Paratylenchus*, *Mesorhabditis*, *Protorhabditis*, *Eucephalobus*, *Heterocephalobus*, *Acrobeles*, *Pseudoaulolaimus*, *Alaimus*, *Ditylenchus*, *Aphelenchus*, *Aphelenchoides*, *Thonus*, *Epidorylaimus* and *Microdorylaimus*, represented common groups. *Eudorylaimus* was a rare group. Over the 2-year field period, the composition of soil nematode communities was essentially uniform in the transgenic insect-resistant cotton fields and the non-transgenic cotton field (Fig. 2). In brief, the soil nematode communities in the 3 fields did not differ significantly.

**Table 2:** Composition and abundance (individual/100 g dry soil) of the soil nematodes extracted from different samples from 3 cotton fields during 2009–2010. *P_i_* denotes the proportion of soil nematode individuals in each treatment.

Trophic groups	Genus	Treat-ment	2009				2010				Total	Percentageof total	Averageabundance
			Seedling	Budding	Boll forming	Boll opening	Seedling	Budding	Bollforming	Bollopening			
Plant parasites	*Tylenchus*	CK	0.31	0.71	1.30	2.40	1.85	0.51	1.10	1.99	10.17	4.2%	
		T-1	0.00	0.34	2.02	2.10	1.47	0.29	1.39	1.54	9.15	3.7%	4.0%
		T-2	0.54	0.00	2.29	1.93	1.47	0.51	0.69	1.73	9.17	3.9%	
	*Filenchus*	CK	2.90	1.47	2.72	1.95	3.08	1.30	3.16	3.28	19.86	8.2%	
		T-1	2.43	1.41	1.55	2.99	2.93	1.67	3.22	3.12	19.32	7.9%	8.2%
		T-2	2.86	1.49	2.78	2.48	3.12	1.54	2.71	3.04	20.01	8.5%	
	*Psilenchus*	CK	0.75	0.51	0.00	0.00	0.00	0.00	0.00	0.00	1.26	0.5%	
		T-1	1.66	0.57	0.00	0.00	0.00	0.29	0.29	0.00	2.80	1.1%	1.1%
		T-2	0.00	0.31	0.59	0.96	0.98	0.00	0.29	0.85	3.97	1.7%	
	*Helicotylenchus*	CK	1.95	1.55	4.28	3.99	1.95	2.48	1.67	2.66	20.54	8.5%	
		T-1	2.39	2.08	4.22	4.12	1.99	2.37	2.04	2.30	21.51	8.8%	8.6%
		T-2	2.68	1.56	3.82	4.29	1.67	2.27	1.79	2.12	20.20	8.6%	
	*Pratylenchus*	CK	2.80	1.81	2.91	2.17	1.54	1.54	1.20	2.20	16.17	6.7%	
		T-1	2.25	1.20	2.77	2.21	1.39	1.39	1.39	2.20	14.79	6.0%	6.4%
		T-2	2.94	1.81	2.65	1.93	1.10	1.39	1.30	1.90	15.01	6.4%	
	*Paratylenchus*	CK	1.52	1.21	0.00	1.72	0.00	0.00	0.51	0.00	4.96	2.1%	
		T-1	2.67	1.41	1.13	1.99	0.00	0.00	0.69	0.00	7.90	3.2%	2.9%
		T-2	2.43	1.10	1.94	2.15	0.69	0.00	0.00	0.00	8.31	3.5%	
	*Longidorella*	CK	0.29	0.98	0.55	1.25	0.85	0.00	0.00	0.00	3.92	1.6%	
		T-1	0.00	0.93	1.05	1.48	0.69	0.00	0.51	0.29	4.95	2.0%	1.7%
		T-2	0.30	0.31	0.54	0.31	0.98	0.00	0.29	0.98	3.71	1.6%	
Bacterivores	*Mesorhabditis*	CK	1.66	1.47	2.74	2.61	0.85	0.85	0.29	0.85	11.31	4.7%	
		T-1	2.33	1.33	2.78	2.59	1.20	0.00	0.69	0.00	10.92	4.5%	4.9%
		T-2	2.18	1.47	2.76	2.88	1.30	0.29	0.51	1.47	12.86	5.5%	
	*Protorhabditis*	CK	2.09	1.00	1.89	3.32	1.61	1.47	2.20	1.95	15.53	6.4%	
		T-1	2.05	0.93	2.60	2.49	1.61	1.30	1.95	1.73	14.65	6.0%	6.2%
		T-2	1.80	1.12	1.70	2.74	1.90	1.85	1.39	1.67	14.16	6.0%	
	*Eucephalobus*	CK	0.55	0.98	1.09	1.17	1.20	0.85	1.47	0.69	8.01	3.3%	
		T-1	0.30	2.37	1.66	2.31	1.10	1.30	1.39	1.20	11.64	4.8%	3.6%
		T-2	0.00	0.72	0.35	0.41	0.69	1.39	1.20	1.67	6.43	2.7%	
	*Heterocephalobus*	CK	1.60	1.62	1.69	0.69	2.77	2.48	1.61	2.16	14.62	6.1%	
		T-1	2.25	1.83	0.42	0.69	2.54	2.46	1.67	2.16	14.04	5.7%	5.8%
		T-2	1.35	1.43	0.00	1.11	2.59	2.51	1.99	1.99	12.97	5.5%	
	*Acrobeles*	CK	1.94	0.87	1.30	0.81	0.29	0.51	0.00	0.00	5.72	2.4%	
		T-1	1.25	0.82	1.99	1.04	0.00	0.00	0.29	0.00	5.38	2.2%	2.0%
		T-2	1.44	0.29	0.35	0.00	0.51	0.00	0.51	0.00	3.10	1.3%	
	*Acrobeloides*	CK	2.85	2.83	2.35	2.47	2.75	2.40	2.98	3.28	21.92	9.1%	
		T-1	2.89	3.32	0.42	2.54	2.85	2.34	3.19	3.39	20.94	8.6%	9.0%
		T-2	2.68	2.54	1.95	2.52	2.83	2.30	3.26	3.50	21.58	9.2%	
	*Panagrolaimus*	CK	0.29	0.30	1.31	1.25	0.00	0.00	0.00	0.51	3.66	1.5%	
		T-1	0.53	0.34	1.44	0.69	0.00	0.00	0.29	0.29	3.58	1.5%	1.5%
		T-2	0.00	0.87	0.00	0.00	0.51	0.29	0.51	1.20	3.38	1.4%	
	*Plectus*	CK	0.72	0.87	1.09	0.00	0.00	0.29	0.00	0.00	2.97	1.2%	
		T-1	1.14	0.95	1.06	0.69	0.00	0.00	0.51	0.00	4.35	1.8%	1.5%
		T-2	1.16	0.87	0.59	0.00	0.51	0.00	0.29	0.00	3.42	1.5%	
	*Chronogaster*	CK	0.31	0.29	0.00	1.08	0.00	0.00	0.51	0.00	2.19	0.9%	
		T-1	0.00	0.31	1.06	0.69	0.29	0.85	0.69	0.00	3.89	1.6%	1.1%
		T-2	0.30	0.00	0.00	0.00	0.00	0.69	0.29	0.69	1.97	0.8%	
	*Pseudoaulolaimus*	CK	0.29	0.00	0.00	0.00	1.73	2.12	1.73	0.85	6.73	2.8%	
		T-1	0.30	0.00	0.86	0.00	0.98	1.85	1.39	0.29	5.66	2.3%	2.5%
		T-2	0.00	0.00	0.00	0.00	1.10	1.73	1.47	0.98	5.28	2.3%	
	*Alaimus*	CK	1.25	0.87	1.39	1.90	2.54	1.54	1.10	1.61	12.20	5.1%	
		T-1	1.43	1.20	1.05	1.92	2.54	1.47	1.30	0.98	11.90	4.9%	4.9%
		T-2	1.44	1.32	0.57	0.92	2.12	1.47	1.73	1.30	10.88	4.6%	
Fungivores	*Ditylenchus*	CK	0.74	1.21	1.09	0.00	0.51	0.69	0.85	0.00	5.10	2.1%	
		T-1	0.73	1.17	0.74	1.96	0.29	0.51	1.20	0.00	6.61	2.7%	2.4%
		T-2	0.54	0.00	0.99	2.18	0.51	0.00	1.20	0.29	5.71	2.4%	
	*Aphelenchus*	CK	1.14	1.39	1.96	1.79	0.00	0.00	0.00	0.00	6.27	2.6%	
		T-1	1.85	2.01	1.91	1.24	0.00	1.10	0.00	0.00	8.10	3.3%	3.3%
		T-2	2.05	2.74	2.18	1.63	0.00	0.85	0.00	0.00	9.46	4.0%	
	*Aphelenchoides*	CK	2.79	2.34	1.30	1.75	1.67	1.14	0.98	0.69	12.68	5.3%	
		T-1	2.60	2.84	1.28	1.55	1.47	0.29	0.29	0.29	10.59	4.3%	4.9%
		T-2	2.40	3.23	1.40	2.38	1.10	0.85	0.00	0.29	11.65	5.0%	
	*Dorylaimoides*	CK	0.87	0.00	0.80	0.69	0.29	0.00	0.00	0.00	2.64	1.1%	
		T-1	0.88	0.00	0.00	0.00	0.00	0.00	0.29	0.00	1.17	0.5%	1.0%
		T-2	1.79	0.69	0.81	0.00	0.00	0.00	0.00	0.00	3.30	1.4%	
Predators	*Mesodorylaimus*	CK	0.29	0.00	1.24	1.09	0.00	0.00	0.29	0.00	2.91	1.2%	
		T-1	0.72	0.00	1.31	0.00	0.29	0.29	0.98	0.00	3.59	1.5%	1.2%
		T-2	0.55	0.00	0.00	0.00	0.29	0.29	0.51	0.69	2.33	1.0%	
	*Thonus*	CK	0.31	1.54	1.54	1.38	0.85	0.29	0.98	1.47	8.36	3.5%	
		T-1	0.31	2.30	0.71	1.46	0.69	0.51	0.85	1.10	7.92	3.2%	3.3%
		T-2	0.72	1.42	1.72	1.09	0.29	0.98	1.10	0.51	7.83	3.3%	
Omnivores	*Eudorylaimus*	CK	0.31	0.00	1.09	0.85	0.00	0.00	0.00	0.00	2.25	0.9%	
		T-1	0.00	0.00	0.76	1.19	0.51	0.00	0.29	0.00	2.75	1.1%	0.9%
		T-2	0.31	0.29	0.54	0.00	0.29	0.00	0.00	0.29	1.72	0.7%	
	*Epidorylaimus*	CK	1.96	1.31	0.00	0.00	0.69	0.29	1.47	1.39	7.11	2.9%	
		T-1	1.52	0.34	0.00	0.00	0.29	1.10	1.30	0.98	5.52	2.3%	2.5%
		T-2	1.85	0.69	0.00	0.00	0.85	0.85	1.10	0.29	5.63	2.4%	
	*Microdorylaimus*	CK	0.72	0.86	1.54	0.00	2.04	1.10	1.39	1.67	9.32	3.9%	
		T-1	0.54	1.62	0.41	1.50	1.95	0.98	0.00	1.54	8.54	3.5%	3.5%
		T-2	0.72	1.12	0.59	0.00	1.73	0.98	0.69	1.20	7.05	3.0%	
	*Aporcelaimellus*	CK	0.26	0.14	0.49	0.42	6.06	0.36	0.20	0.69	0.60	1.1%	
		T-1	0.41	0.00	0.49	0.35	4.72	0.15	0.00	0.69	0.49	1.0%	1.2%
		T-2	0.29	0.42	0.68	0.35	7.35	0.01	0.60	0.43	0.09	1.5%	

### Effect of Transgenic Insect-resistant Cotton on the Number of Soil Nematodes

**Figure 3:**
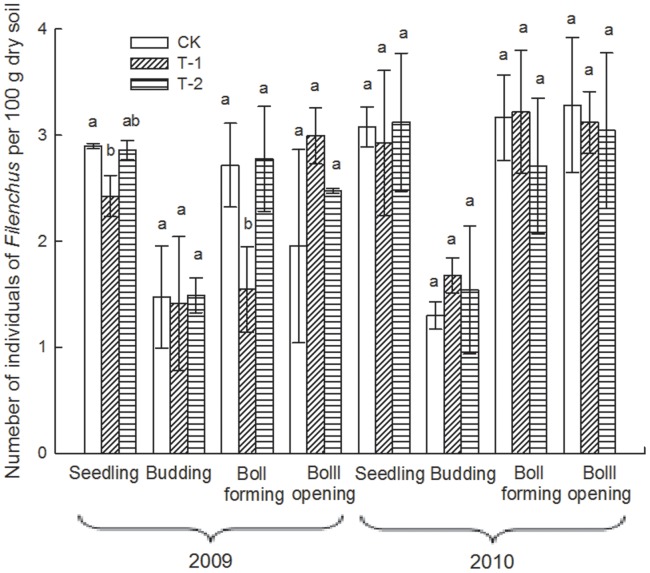
Number of *Filenchus* in 3 cotton fields at different sampling times. Error bars indicate standard errors (n = 3). Different letters above bars denote a statistically significant difference between the means of the fields.

**Figure 4:**
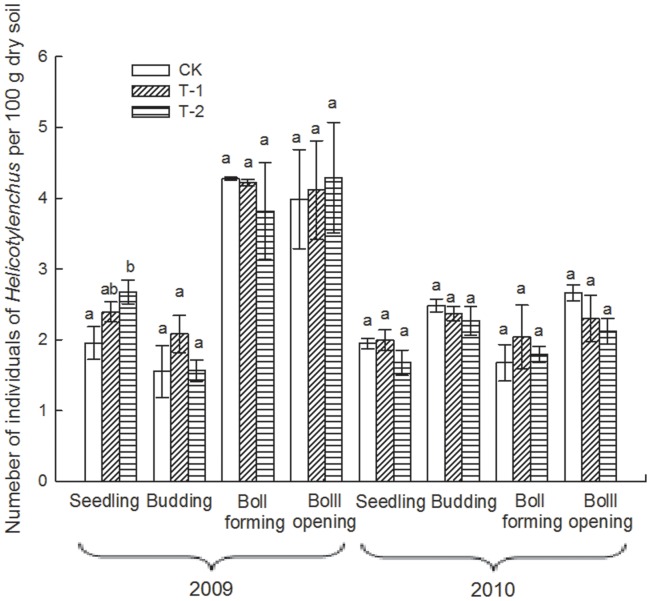
Number of *Helicotylenchus* in 3 cotton fields at different sampling times. Error bars indicate standard errors (n = 3). Different letters above bars denote a statistically significant difference between the means of the fields.

**Figure 5:**
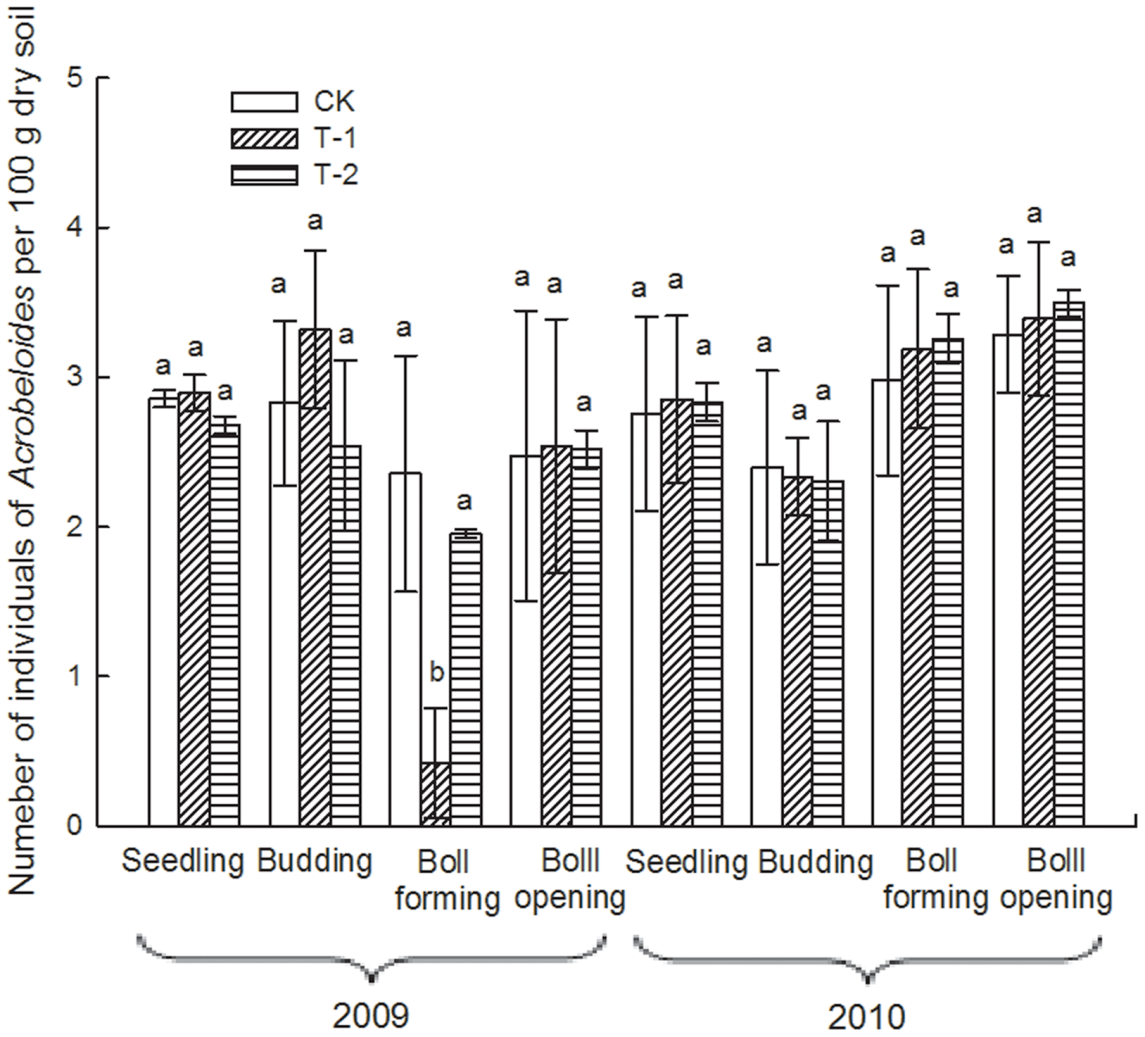
Number of *Acrobeloides* in 3 cotton fields at different sampling times. Error bars indicate standard errors (n = 3). Different letters above bars denote a statistically significant difference between the means of the fields.

Due to the particularly arid conditions occurring throughout 2010, the number of total soil nematodes for the 4 sampling times in 2010 was obviously less than that for the 2009 samples (Table 2). During the 2-year sampling period, the number of total soil nematodes and the most abundant nematodes, such as *Filenchus, Helicotylenchus* and *Acrobeloides*, in each cotton field varied significantly among different sampling times (i.e., different growth stages of the plants; *p*<0.01; Table 3) but did not differ significantly overall among the 3 cotton fields (*p*>0.05; Table 3). At the seedling and boll-forming stages in 2009, the number of *Filenchus* in T-1 was significantly lower than that in CK (*p*<0.05; Fig. 3). At the seedling stage in 2009, the number of *Helicotylenchus* in T-2 was significantly greater than that in CK (*p*<0.05; Fig. 4). At boll-forming stage in 2009, the number of *Acrobeloides* in T-1 was significantly lower than that in CK and T-2 (*p*<0.05; Fig. 5). For the other sampling times, the numbers of *Filenchus, Helicotylenchus* and *Acrobeloides* did not vary significantly among the 3 fields (*p*>0.05; Figs. 3–5).

**Table 3:** Generalized Linear Mixed Model (GLMM) results for overall effects on the numbers of soil nematodes.

Effects	Degrees of freedom	F Value	*p* Value
Total soil nematodes			
Treatments	2;14	0.69	0.52
Sampling time	7;14	24.53	0.00
*Filenchus*			
Treatments	2;14	0.12	0.88
Sampling time	7;14	9.98	0.01
*Helicotylenchus*			
Treatments	2;14	1.00	0.39
Sampling time	7;14	49.47	0.00
*Acrobeloides*			
Treatments	2;14	0.18	0. 84
Sampling time	7;14	5.34	0.00

### Effect of Transgenic Insect-resistant Cotton on Soil Nematode Trophic Groups

In all, 26–57% of the nematodes were bacterial feeders, 24–47% plant parasites, 1–25% fungal feeders, 3–15% omnivores and 1–8% predators. The feeding-type composition was relatively constant throughout the 2-year survey, with average proportions of 41–44% bacterial feeders, 32–34% plant parasites, 11–13% fungal feeders, 9–11% omnivores and 2% predators (Table 4). These values showed no significant differences in feeding-type composition among the 3 cotton fields at different sampling times (*p*>0.05; one-way ANOVA). During the 2 years (2009 and 2010), the proportions of bacterial feeders, fungal feeders, plant parasites, omnivores and predators in each cotton field varied significantly among sampling times (*p*<0.05; Table 5) but did not differ significantly overall among the 3 cotton fields (*p*>0.05; Table 5).

**Table 4:** Changes in the composition of nematode trophic groups (%) among different cotton fields at different sampling times for the 2-year survey (mean±SE).

Trophic groups	Treatment	2009				2010			
		Seedling	Budding	Boll forming	Boll opening	Seedling	Budding	Boll forming	Boll opening
Plant parasites	CK	31.6±0.8^a^	29.5±2.1^a^	31.6±4.7^a^	37.1±2.9^a^	31.3±2.6^a^	26.4±3.7^a^	28.9±2.4^a^	36.1±6.7^a^
	T-1	32.3±1.9^a^	25.1±1.7^a^	36.2±3.8^a^	37.1±1.3^a^	30.7±3.2^a^	26.9±2.4^a^	32.8±3.9^a^	40.4±3.8^a^
	T-2	33.2±0.2^a^	24.0±2.3^a^	46.4±9.7^a^	43.5±7.6^a^	34.0±4.5^a^	23.9±4.3^a^	27.4±2.8^a^	36.7±4.2^a^
Bacterial feeders	CK	40.8±4.9^a^	39.6±11.7^a^	40.0±6.3^a^	42.1±5.8^a^	46.5±4.6^a^	56.5±7.6^a^	44.9±1.2^a^	42.3±4.7^a^
	T-1	41.0±5.8^a^	42.4±5.2^a^	43.6±8.5^a^	39.0±3.6^a^	47.6±5.5^a^	51.7±9.4^a^	46.0±2.7^a^	42.9±6.9^a^
	T-2	34.9±10.0^a^	38.8±9.6^a^	26.3±13.7^a^	32.7±7.4^a^	47.8±7.3^a^	52.5±3.7^a^	51.0±4.5^a^	50.0±5.6^a^
Fungal feeders	CK	16.7±5.9^a^	17.7±2.4^a^	13.9±3.2^a^	11.7±3.2^a^	8.4±2.7^a^	8.3±1.4^a^	6.9±1.3^a^	2.5±0.6^a^
	T-1	17.1±4.4^a^	19.0±2.3^a^	11.2±3.1^a^	11.8±2.3^a^	6.4±3.7^a^	8.5±1.7^a^	6.1±2.7^a^	1.2±0.7^a^
	T-2	19.2±7.3^a^	24.3±6.1^a^	17.1±4.7^a^	19.2±3.5^a^	5.5±1.6^a^	7.1±2.7^a^	4.7±1.9^a^	2.0±1.3^a^
Predators	CK	1.8±0.3^a^	5.5±1.5^a^	7.5±1.9^a^	6.8±2.1^a^	2.9±1.2^a^	1.3±0.9^a^	4.8±2.1^a^	5.2±1.8^a^
	T-1	2.9±0.2^a^	7.3±1.3^a^	5.7±2.2^a^	3.6±1.5^a^	3.6±1.8^a^	3.6±1.5^a^	6.3±1.8^a^	4.7±2.2^a^
	T-2	3.6±1.2^a^	5.2±2.8^a^	5.5±1.9^a^	3.4±1.7^a^	2.0±0.5^a^	5.3±2.8^a^	6.2±2.7^a^	4.2±1.7^a^
Omnivores	CK	9.0±1.3^a^	7.8±3.2^a^	7.1±2.7^a^	2.3±1.1^a^	11.0±2.5^a^	7.6±2.3^a^	14.5±1.9^a^	13.9±5.1^a^
	T-1	6.7±3.5^a^	6.2±1.8^a^	3.3±1.9^a^	8.4±3.7^b^	11.8±1.3^a^	9.3±3.8^a^	8.8±2.1^a^	10.8±2.3^a^
	T-2	9.0±0.3^a^	7.7±1.4^a^	4.8±2.3^a^	1.3±2.8^a^	10.7±2.9^a^	11.2±3.7^a^	10.7±2.9^a^	7.1±3.8^a^

Mean values and standard error of 3 replicates are presented. Use of the same letter as a superscript indicates that variable means did not differ significantly among different treatments at each sampling date (*p*>0.05).

**Table 5:** Generalized Linear Mixed Model (GLMM) results for the overall effects on soil nematode trophic groups.

Effects	Degrees of freedom	F Value	*P* Value
Plant parasites			
Treatments	2;14	0.62	0.55
Sampling time	7;14	6.28	0.00
Bacterial feeders			
Treatments	2;14	0.85	0.45
Sampling time	7;14	4.56	0.01
Fungal feeders			
Treatments	2;14	2.07	0.16
Sampling time	7;14	15.77	0.00
Predators			
Treatments	2;14	0.12	0.89
Sampling time	7;14	4.24	0.01
Omnivores			
Treatments	2;14	0.78	0.48
Sampling time	7;14	1.99	0.13

### Effect of Transgenic Insect-resistant Cotton on the Ecological Indices of Soil Nematodes

**Figure 6:**
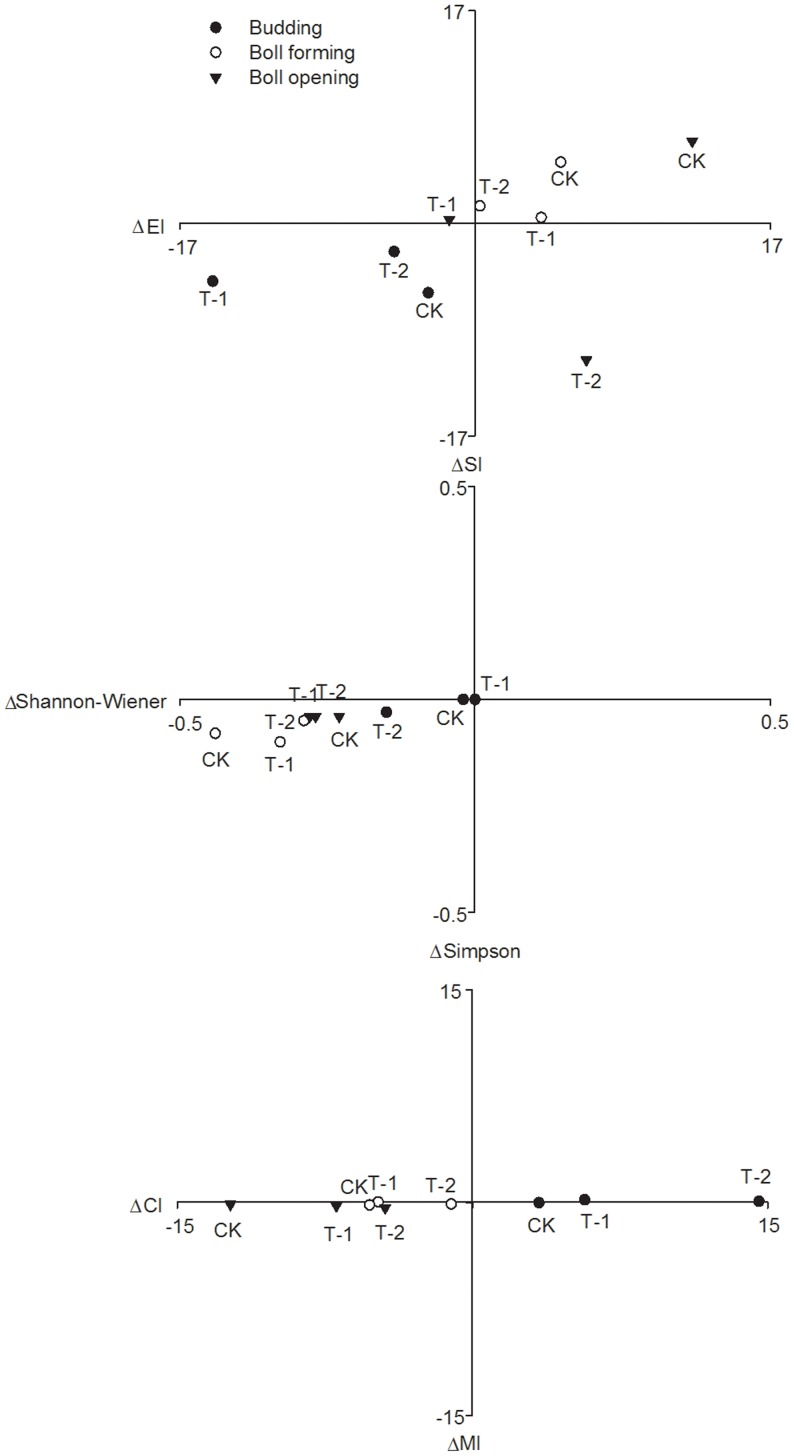
Changes in the values of ecological indices for soil nematode faunal analysis relative to seedling stage among different cotton fields.

The initial values of the Shannon–Wiener, Simpson, EI, SI, CI and MI ecological indices were obtained from the analysis of the nematode fauna at the seedling stage. Differences (Δ) from these initial values were obtained for each cotton field at the budding, boll-forming and boll-opening stages (Fig. 6). A univariate general linear model analysis suggested that these differences in the ecological indices varied significantly overall among different sampling times (*p*<0.05) but did not differ significantly overall among the 3 cotton fields (*p*>0.05). A further analysis used a one-way ANOVA with a priori contrasts to compare these ecological indices for the conventional cotton field with the corresponding indices for the 2 transgenic fields but detected no differences in the values of the ecological indices of the transgenic fields relative to the conventional cotton field at the 8 sampling times.

### Principal Component Analysis of Soil Nematode Composition in Cotton Fields

**Figure 7:**
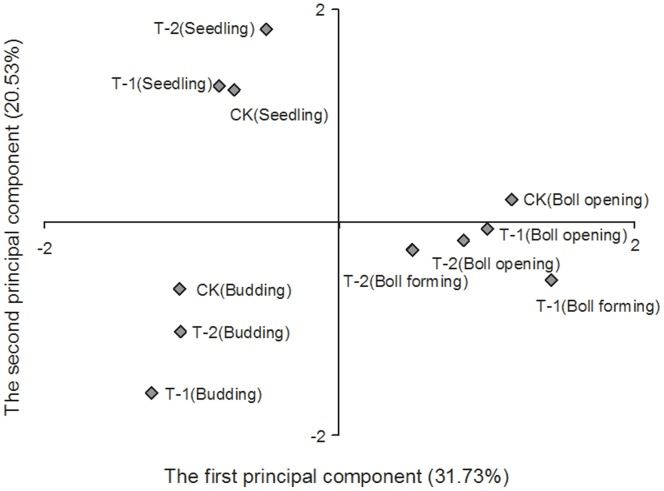
Factor loading graph of principal component analysis of soil nematode generic composition in cotton fields at different sampling times.

Five principal components were selected for the analysis based on a cumulative contribution rate of 85% for the principal components extracted. The contribution rates of the first 2 principal components were 31.73% and 20.53%, respectively (Fig. 7). Different sampling times showed a distinct separation along the principal component axes, whereas different fields formed a cluster at the same sampling time. The first principal component axis clearly separated the budding, boll-forming and boll-opening stages. The second principal component axis clearly distinguished the seedling and budding stages (Fig. 7).

## Discussion

With the cultivation of more varieties of transgenic insect-resistant plants and their large-scale planting, environmental impact monitoring after commercial release has attracted increasing attention from the scientific community [37], [49]–[51]. High population densities and large numbers of species of nematodes occur in almost all soils. In this study, potential effects on soil nematodes at the community level were monitored during a 2-year survey to assess the environmental risks associated with transgenic fields planted with transgenic insect-resistant cotton for more than 10 years relative to the risks associated with a field planted with non-transgenic cotton. Based on the nematode communities examined at the genus level, the overall findings of the 2-year field study showed that the community structure of the soil nematodes was similar in the 3 cotton fields and that the most abundant common groups of soil nematodes all included *Helicotylenchus*, *Filenchus* and *Acrobeloides*. In soil planted with *Bt* maize and *Bt* eggplant expressing the Cry1Ac and Cry3Bb1 proteins, respectively, no effects were found on nematode community structure [32], [52]. However, a distinct shift in community structure, i.e., a significantly higher proportion of mycophagous nematodes and a lower proportion of phytophagous nematodes, were observed in soil planted with *Bt* canola relative to soil planted with the respective non-*Bt* isoline [53]. In a study of the effects of Mon88017 cultivation on the community structure of the indigenous soil-inhabiting nematodes, no significant differences in the generic composition of the nematodes was found between a *Bt* maize plot and non-*Bt* maize plots, but a significant shift in generic composition was found at the final sampling date [48]. In a few cases, the studies cited reported some differences in the nematode communities among the different treatments at certain sampling times, but no consistent significant differences were found over the entire sampling period, and these studies only covered a short sampling time period. The current study observed no significant differences in soil nematode communities between fields of transgenic insect-resistant cotton and a field of non-transgenic cotton, and the study indicated that long-term cultivation of transgenic insect-resistant cotton had no significant impact on the composition and community structure of soil nematodes in agricultural soils.

Laboratory and field studies have shown no consistent effects of transgenic insect-resistant maize on the number of soil nematodes relative to non-*Bt* maize [31], [33], [34]. The cultivation of *Bt* maize expressing the Cry1Ab protein significantly decreased the number of soil nematodes at 3 different field sites relative to non-*Bt* maize, whereas a study conducted in a greenhouse showed no toxic effects of the Cry1Ab protein on populations of nematodes. In contrast, both the sampling site and the time had greater significant influences on the population density of soil nematodes than that of the maize lines [35], [36]. Based on a GLMM analysis of data collected over 2 years of sampling, our results indicated that the number of functional guilds in the 3 cotton fields showed significant seasonal variation (necessarily following the progression of different cotton growth stages). However, the effect of long-term cultivation of transgenic insect-resistant cotton (T-1 and T-2) on the number of total soil nematodes and on certain dominant groups, such as *Filenchus, Helicotylenchus* and *Acrobeloides*, was not significant relative to the values found for conventional cotton cultivation. In only a few instances in our study did we find significant differences in the numbers of soil nematodes between fields of different cultivars. No consistent trend was found over the 2 study years. This general finding is in accord with work by other teams in other regions and on a variety of crops [31], [33], [35], [36], [54], [55].

Parasitic nematodes can cause considerable economic damage worldwide to many types of crops, including cotton in the major growing areas of certain countries [56], [57]. Several plant parasitic nematodes, such as *Helicotylenchus*, *Tylenchorhynchus*, *Tylenchus*, *Pratylenchus* and *Filenchus*, have been detected in the cotton fields of north China [58]–[60]. Our study found that *Filenchus*, *Helicotylenchus*, *Tylenchus* and *Pratylenchus* were the principal genera of plant parasitic nematodes in the 3 cotton fields. These results were consistent with the findings of other studies conducted in China. Certain genera, e.g., *Meloidogyne*, *Rotylenchulus* and *Belonolaimus*, that are known to infest cotton were not detected in the soils of the 3 cotton fields. The principal explanation for this result might be that these nematodes are of concern in the United States, India, Pakistan, Egypt and Brazil but not in China [61] and are seldom separated from soils sampled in the cotton-growing region of north China [58]–[60]. Moreover, these nematodes primarily parasitize the root tissues of the plant and are very rare in the soil around the plant roots. Finally, outbreaks of cotton nematode diseases have not been recorded in the 3 cotton fields for more than 20 years. Therefore, the nematode communities collected from the soil samples in the study were typical of China.

Functional analyses and indices such as those used in ecological studies have proven relatively useful in detecting the true effects of the cultivation of transgenic plants [18], [34], [62]. Over 2 years of sampling, we observed strong and significant seasonal variations in the ecological indices of the soil nematodes collected in all 3 cotton fields, in parallel with the distinct growth stages of cotton. However, no statistically significant effects of the long-term cultivation of transgenic insect-resistant cotton (in fields T-1 and T-2) on the ecological indices of the soil nematodes were evident relative to the non-*Bt* cotton (field CK). This result agreed with those reported by Manachini and Lozzia (2002) and Höss et al. (2011), who found that *Bt* maize expressing the Cry1Ab and Cry3Bb1 toxins had no significant effects on the diversity of soil nematodes [32], [34].

The accumulation of *Bt* protein in soil has long been posited as one of the main putative mechanisms for the effects of transgenic *Bt* plants on soil organisms [9], [68]–[70]. However, many studies have demonstrated that Cry proteins degrade rapidly in soil under laboratory conditions [70]–[72] and, hence, are unlikely to accumulate or persist in fields where *Bt* crops have been planted for years [73]–[75]. For example, the content of Cry1Ab protein was above the detection limit of an ELISA test in only half of the soil samples obtained from transgenic plots, ranging from 0.19 to 1.31 ng g^−1^ dry weight [34]. To address this possible but unlikely occurrence at our study site, we determined the residual levels of Cry1Ac protein in the soils of T-1 and T-2 using a QualiplateTM kit for Cry1Ab/Cry1Ac (EnviroLogix, USA) and found extremely low levels of Cry1Ac protein, below the quantitative limit of the kit [76]. These results agreed with the findings of others [73]–[75], indicating that Cry1Ab/c protein did not accumulate in cambisol soil with prolonged planting of transgenic cotton.

There was also no indication that the community structure of the soil nematodes was influenced by indirect effects of transgenic proteins, such as Cry1Ab and Cry1Ac, via the food web. Microbial communities such as bacteria, actinomycetes and fungi and soil invertebrate communities such as Collembola, Opisthophora and Acarina, which were studied in the same cotton fields investigated in the present study, showed no significant differences in abundance and diversity between transgenic insect-resistant cotton and non-*Bt* cultivars [76], [77]. These findings are consistent with the results of another study examining the effect of transgenic insect-resistant plants on microbial populations [75], [78], [79] and other soil organisms, such as Collembola, mites and earthworms [62], [80]–[82]. Clearly, soil organisms are not impacted or are only slightly impacted by the cultivation of transgenic insect-resistant cotton.

Many studies on the effects of pesticides (primarily nematicides and insecticides) on total nematode abundance and feeding groups under field or semi-field conditions have been conducted. The majority of these studies have indicated no negative effects of pesticides such as malathion, imicyafos and carbofuran on nematodes [63]–[66]. In one study, a decrease in abundance was observed as an effect of pesticides such as nemacur [67]. In the current study, the same spraying dose of chemical pesticides (i.e., avermectin and halfenprox) used to control insects with piercing-sucking mouthparts, such as the red spider, was applied to the 3 fields. However, the applications of chemical pesticides (i.e., methamidophos and cypermethrin) used to control Lepidoptera, such as the cotton bollworm, in the 2 transgenic cotton fields were fewer in number than those applied to the conventional cotton field (Fig. 1). Therefore, the differences in the spraying of chemical pesticides to control Lepidoptera in the 3 cotton fields might be another important influence on the soil nematode community, and the total pesticide applications could mask the effects of different cotton lines in the present study, or the effects of transgenic cotton lines on the soil nematode community might be smaller than those of the pesticide regimes.

Transgenic plants must be monitored for environmental risk after being commercially released [50], [83]. The risks potentially posed by transgenic plants, especially *Bt* crops, to the environment have been extensively assessed worldwide over the past 10 years, and no scientific evidence has shown that the cultivation of *Bt* crops has caused sustained environmental harm to communities of soil organisms, such as nematodes, earthworms, collembolans or mites [33], [35], [50], [62]. However, the soil environment is a very complex ecosystem in which many factors affect the soil biota. In field studies, high variability in biotic parameters is inherent and usually present, and this variability must be considered seriously if the ecological risks posed by transgenic plants are to be monitored. Moreover, the soil biota may be strongly stressed as a result of the influence of environmental factors (e.g., pH, salinity, redox potential, vegetation and water-holding capacity), which may cause higher or lower levels of sensitivity to transgenic plants [23], [40], [56]. Therefore, it is necessary to continue monitoring the effects of transgenic plants on the soil ecosystem in different environments and to define the ecological significance of the planting of transgenic crops.
